# Development and Optimization of an Unbiased, Metagenomics-Based Pathogen Detection Workflow for Infectious Disease and Biosurveillance Applications

**DOI:** 10.3390/tropicalmed8020121

**Published:** 2023-02-15

**Authors:** Kyle Parker, Hillary Wood, Joseph A. Russell, David Yarmosh, Alan Shteyman, John Bagnoli, Brittany Knight, Jacob R. Aspinwall, Jonathan Jacobs, Kristine Werking, Richard Winegar

**Affiliations:** 1 MRIGlobal, 425 Dr. Martin Luther King Jr. Blvd, Kansas City, MO 64110, USA; 2 MRIGlobal, 65 West Watkins Mill Road, Gaithersburg, MD 20850, USA

**Keywords:** biosurveillance, host depletion, infectious disease, next-generation sequencing

## Abstract

Rapid, specific, and sensitive identification of microbial pathogens is critical to infectious disease diagnosis and surveillance. Classical culture-based methods can be applied to a broad range of pathogens but have long turnaround times. Molecular methods, such as PCR, are time-effective but are not comprehensive and may not detect novel strains. Metagenomic shotgun next-generation sequencing (NGS) promises specific identification and characterization of any pathogen (viruses, bacteria, fungi, and protozoa) in a less biased way. Despite its great potential, NGS has yet to be widely adopted by clinical microbiology laboratories due in part to the absence of standardized workflows. Here, we describe a sample-to-answer workflow called PanGIA (Pan-Genomics for Infectious Agents) that includes simplified, standardized wet-lab procedures and data analysis with an easy-to-use bioinformatics tool. PanGIA is an end-to-end, multi-use workflow that can be used for pathogen detection and related applications, such as biosurveillance and biothreat detection. We performed a comprehensive survey and assessment of current, commercially available wet-lab technologies and open-source bioinformatics tools for each workflow component. The workflow includes total nucleic acid extraction from clinical human whole blood and environmental microbial forensic swabs as sample inputs, host nucleic acid depletion, dual DNA and RNA library preparation, shotgun sequencing on an Illumina MiSeq, and sequencing data analysis. The PanGIA workflow can be completed within 24 h and is currently compatible with bacteria and viruses. Here, we present data from the development and application of the clinical and environmental workflows, enabling the specific detection of pathogens associated with bloodstream infections and environmental biosurveillance, without the need for targeted assay development.

## 1. Introduction

The prevalence of infectious diseases impacts public health and security, while also affecting global economies and political systems [1]. Progress has been made towards eradicating some diseases, such as polio and malaria [2], and there has been a reduction in the number of annual deaths associated with these diseases [3]. However, emerging infectious diseases and the threat of bioterrorism agents pose challenges to reducing the global burden of these diseases [4]. Thus, identifying and characterizing these known and emerging etiological agents remains a crucial aspect of the global response to infectious diseases. Traditionally, infectious disease detection has relied on culture-based methods for identifying bacterial, viral, and fungal pathogens. However, the utility of culture-based methods can be limited with slow-growing organisms, have fastidious growth conditions, or pose a biohazard risk to laboratory workers. Additionally, the positive predictive value of culture methods varies according to the organism, complicating the determination of true bacteremia versus contamination [5].

Alternative methods, which are often used in concert with pathogen detection include the use of microscopy, serology, and a variety of molecular methods. Technological advancements have resulted in the development of molecular diagnostic tools enabling faster turnaround times, direct detection from a clinical or environmental sample, and the ability to provide high-confidence detection [6,7,8]. Most molecular diagnostic tools utilize polymerase chain reaction (PCR), a nucleic acid amplification test (NAAT), for pathogen identification based on detecting specific nucleic acid sequences. Despite the rapid turnaround time and the ease of use, most NAATs are limited to the detection of between 1–30 pathogens, with the majority targeting a single pathogen. In addition, NAATs often fail to detect new pathogen variants that have evolved due to mutations in the genome or acquisition of new genetic elements through horizontal gene transfer [9].

Next-generation sequencing (NGS) can allow pathogen detection in scenarios where traditional methods have generated negative or inconclusive results [10,11,12,13,14]. The ability of NGS to sequence millions of library fragments in parallel, with greater depth, is a significant improvement over traditional Sanger sequencing technologies. Whole genome sequencing allows for investigating an organism’s entire genome. It can utilize specific markers for pathogen detection, while targeted NGS utilizes amplicons or hybridization capture techniques to query specific target sequences. While targeted NGS methods offer increased pathogen detection, this approach is also limited by the ability to detect only the targeted pathogens [15]. Alternatively, metagenomic NGS offers the ability for less biased pathogen detection and characterization [13]. Despite the many advantages NGS offers, this technology is not routinely utilized in clinical laboratories due to the lack of standardized protocols; clinical guidelines for optimal use and appropriate result interpretation; and the challenges associated with analyzing complex genomic data [16,17]. This work aimed to develop a non-targeted, metagenomics-based pathogen detection system that meets the operational requirements described in Table 1, addressing the needs for the biosurveillance community.

Developing a standardized workflow that meets these requirements presents challenges. The optimization of sample preparation is crucial for high-quality sequencing results and specific considerations are necessary for different matrices. For example, detecting low-titer pathogens in clinically relevant samples is complicated by a high level of host genetic material [18]. Environmental samples often comprise diverse microbial populations and contain components that interfere with sample processing and sequencing library preparation. Additionally, bioinformatics analysis needs to balance inexpensive, lightweight commercial hardware with the need for speed, accuracy, and high information content.

**Figure 1:**
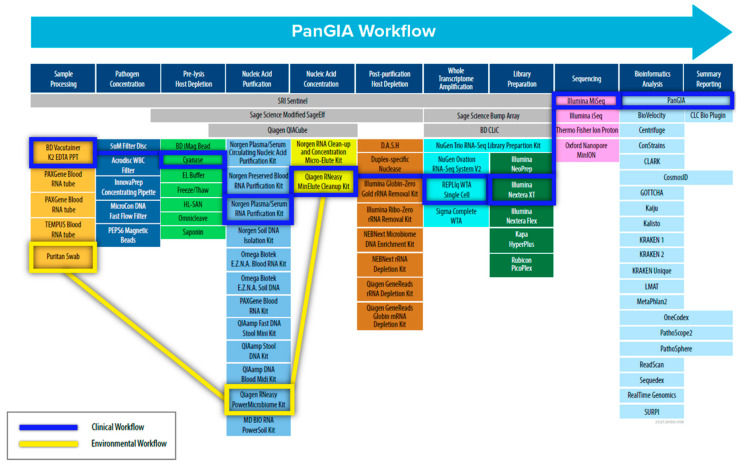
Schematic Diagram of the PanGIA Sample-to-Sequence Workflow. This figure outlines all the technology evaluated during the development of the Clinical and Environmental Sample-to-Sequence Workflows. The individual technologies integrated into the final PanGIA workflow are highlighted in blue for the clinical workflow and yellow for the environmental workflow. Categories that lack a selected technology were not incorporated into the final iteration of the PanGIA Workflow.

To address these challenges, we adopted a “Best-of-Breed” approach to identify, evaluate, and select commercial-off-the-shelf (COTS) technologies and products to use in the PanGIA (Pan-Genomics for Infectious Agents) final workflow (refer to Figure 1). We evaluated 79 technologies for the proposed workflow, encompassing sample pre-processing, pathogen concentration, pre-lysis host depletion, total nucleic acid purification, total nucleic acid concentration, whole transcriptome amplification, post-purification host depletion, library generation, next-generation sequencing, and bioinformatics analysis. The work presented here details our development of a separate clinical workflow (intended for use with clinically relevant matrices such as whole blood, plasma, serum, and saliva.) and environmental workflow (intended for use with environmental samples such as swabs, soil, wastewater, etc.). Our initial development focused on using whole blood for bloodstream infections and environmental swabs in our developed workflow as sample inputs. The work presented here is offered as an unbiased solution for pathogen detection for infectious disease and biosurveillance applications, with future goals of integrating additional relevant sample types.

## 2. Materials and Methods

### 2.1. Best-of-Breed Approach

To develop an unbiased, sample-to-sequence workflow, we performed a best-of-breed analysis to understand what technologies and methods are best suited for system integration. This analysis included identifying, evaluating, and down-selecting candidate technologies for each workflow component that supported the program requirements. Notably, the final workflow had to be compatible with bacteria, DNA virus and RNA viruses. The best-of-breed process included literature reviews, end-user feedback and wet-lab experiments. Due to the many candidate technologies, not all combinations were evaluated with wet-lab experiments. For some components, testing was minimal, and down-selection was based on previous experience with the selected technologies. Priority was given to workflow components critical for achieving high detection sensitivity, including dehosting and total nucleic acid extraction and purification.

### 2.2. Preparation of Quantified Bacterial and Viral Stocks

To simulate infected blood samples, we used stocks of the following pathogen surrogate organisms for spiking material into whole blood and contrived environmental swabs: *Vibrio cholerae*, strain MO45 (BEI# NR-144); *Staphylococcus aureus*, strains FDA S6 (ATCC # 13566) and Seattle 1945 (ATCC# 25923); Venezuelan equine encephalitis (VEE) virus strain TC-83, Yellow Fever vaccine strain 17D, and Modified Vaccinia virus Ankara (MVA). These organisms were chosen to represent different organism classes (gram-negative bacteria, gram-positive bacteria, RNA and DNA viruses). Bacterial stocks were prepared from single colony isolates cultured with appropriate media and incubation periods, as specified in their respective product sheet. Bacterial cells were then pelleted and resuspended in 10% glycerol for long-term storage. Aliquots of bacterial stocks were plated for enumeration. Viral stocks were propagated by cell culture and then enumerated by plaque assays. In addition, contrived samples were spiked with *Escherichia coli* bacteriophage Phi X174 (ATCC 13706) and *Escherichia coli* bacteriophage MS2 (ATCC 15597-B1) at approximately 1e7 PFU/mL as exogenous, internal controls (EICs) for detection of DNA and RNA, respectively.

### 2.3. Preparation of Contrived Samples for Analytical Studies

#### 2.3.1. Contrived Human Blood Samples

Single-donor whole human blood was acquired from Zen-Bio, Inc. (Research Triangle Park, NC, USA) and Bioreclamation IVT (New York, NY, USA). For the analytical studies, 1 mL whole blood samples were spiked with only the EICs (noted in our experiments as a Quality Control Sample or QCS) or 10 µL of a spiking mixture containing EICs and pathogen surrogates, as described above.

#### 2.3.2. Simulated Environmental Surface Samples

Reference Biological Material (RBM), consisting of bulk particulate collected from HVAC return ducting and mechanical chases from buildings located throughout the continental United States, was prepared in-house at MRIGlobal, and used to simulate environmental surface collection samples. To prepare a simulated environmental swab, a Sterile Polyester Tipped Applicator Swab (Puritan^®^ 25-806-1PD) was moistened with 100 μL of phosphate-buffered saline (PBS) and ~0.2 mg of RBM was added to the swab by swirling the moistened swab in a weigh boat containing RBM. For the analytical studies, swabs were spiked with only the EICs or 10 µL of a spiking mixture containing EICs and pathogen surrogates, as described above.

### 2.4. Whole Blood Preprocessing

For each 1-mL spiked whole blood sample, 25 μL of blood was aliquoted into a new 2 mL microcentrifuge tube. The remaining blood volume was centrifuged at 1100 RCF for 10 min in an Eppendorf centrifuge with a swinging bucket rotor. Following centrifugation, the plasma layer (approximately 500 µL) was transferred to the 2 mL microcentrifuge tube containing 25 µL of whole blood. The resulting sample for processing consists of 500 µL plasma +25 µL whole blood. This blood sample processing is necessary for improving the dehosting reaction efficiency while retaining a fraction of the whole blood for processing potential intracellular pathogens.

### 2.5. Pre-Lysis Host Depletion

Cell-free nucleic acids in the plasma fraction were digested with Benzonase^®^ Nuclease Alternative Cyanase™ (Ribosolutions Inc., Cedar Creek, TX, USA). To each sample, 10 µL of 1 M MgCl_2_ and 100 Units of Cyanase were added. Samples were pulse vortexed for 10 s, then incubated at ambient temperature (22 °C) for 10 min. Following incubation, 100 µL of Cyanase Inactivation Resin was added and inverted 20 times. Samples were centrifuged at 500× *g* for 3 min to pellet the inactivation resin before transferring the supernatant.

Several methods were evaluated for differential lysis. EL Buffer (Qiagen, Hilden, Germanyrmany) was mixed with spiked whole blood samples in a 2:1 EL Buffer: blood ratio. For freeze-thaw lysis, whole blood samples were diluted with both water and EL Buffer and then frozen at 20 °C for 20 min and allowed to thaw to room temperature before processing. For treatment with Saponin, 100 µL of a 10% Saponin solution was added to 1 mL of whole blood for a final concentration of 1% Saponin. Reactions were incubated at room temperature for 5 min, followed by inactivation with 2 mL of molecular-grade water.

In addition to Cyanase, we investigated an additional endonuclease: Omnicleave (Epicentre, Madison, WI, USA). For treatment with Omnicleave, 10 µL of 1 M MgCl_2_ and 1000 Units of Omnicleave were added per 1 mL of fractionated blood. Samples were pulse vortexed for 10 s and incubated at room temperature for 10 min prior to nucleic acid extraction.

### 2.6. Total Nucleic Acid (TNA) Extraction

#### 2.6.1. Whole Blood TNA Extraction and Purification

Approximately 500 µL of the dehosted sample was added to a PowerBead (0.1 mm glass) tube (Qiagen catalog #13118-50) with 500 µL Lysis Buffer A. Bead-beating was performed on a Vortex Genie 2 with a vortex adapter for 10 min at maximum speed. Total nucleic acids were purified with Plasma/Serum RNA Purification Midi kit (Norgen Biotek, Thorold, ON, Canada). The manufacturer’s protocol was followed, with minor modifications [19]. All centrifugation steps were increased to 3000× *g* for 3 min and the final elution (200 µL) was performed following column drying (Step 7). The following kits were also evaluated for purification of total nucleic acids from whole blood: QIAamp DNA Blood Kit (Qiagen, Hilden, Germany), PAXgene Blood RNA System (Qiagen, Hilden, Germany), Preserved Blood RNA Purification Kit (Norgen Biotek, Thorold, ON, Canada), RNAgard Blood System (Biomatrica, San Diego, CA, USA), NucleoSpin RNA Blood Midi (MACHEREY-NAGEL GmbH & Co., Duren, Germany), E.Z.N.A.^®^ Blood RNA Midi Isolation (Omega Bio-tek, Norcross, GA, USA).

#### 2.6.2. Environmental TNA Extraction

Environmental forensic swabs were extracted with the RNeasy Power Microbiome Kit (Qiagen, Hilden, Germany) according to the manufacturer’s protocol, excluding the DNase I Removal step [20]. We also evaluated the following kits for environmental forensic swabs: QIAamp Fast DNA Stool Mini kit, QIAamp DNA Blood Mini kit (Qiagen, Hilden, Germany), Soil Total RNA Purification Kit (Norgen Biotek, Thorold, ON, Canada). Each extraction was performed for initial assessments according to the manufacturer’s protocols. When assessing recovery of total nucleic acids from RNA kits, the DNase treatment step was omitted from the protocol, when applicable.

### 2.7. TNA Concentration via MinElute

RNeasy MinElute Cleanup kit (Qiagen, Hilden, Germany) was used to concentrate total nucleic acid (TNA) samples following the extraction of clinical and environmental matrices [21]. TNA contains both DNA and RNA purified from the extraction process. The protocols for 100 µL and 200 µL samples were followed for TNA samples from the environmental and clinical workflows, respectively. Concentrated TNA samples were eluted from the column in approximately 14 µL of RNase-free water. Half was used for Whole Transcriptome Amplification (WTA) and the other half was retained as concentrated TNA. We also evaluated the Norgen RNA Clean-Up and Concentration Micro-Elute Kit to concentrate purified TNA samples. The manufacturer’s protocol was followed for this kit.

### 2.8. Real-Time PCR (qPCR)

PCR master mix was made by combining assay-specific primers and probes with TaqMan^®^ Universal Master Mix II, no UNG (Life Technologies, Carlsbad, CA, USA). SuperScript^®^ III One-Step RT-PCR System with Platinum^®^ Taq DNA Polymerase (Life Technologies) was used to make the master mix, along with assay-specific primers and probes, for the reverse-transcription real-time PCR reactions. All real-time PCR reactions were performed on the CFX 96 platform (Bio-Rad, Hercules, CA, USA).

### 2.9. Post-Purification Host Depletion

The following commercially available ribosomal RNA and microbial enrichment kits were evaluated for this effort: NEBNext Microbiome DNA enrichment kit (New England Biolabs Inc., Ipswich, MA, USA), NEBNext^®^ rRNA Depletion Kit (Human/Mouse/Rat) (New England Biolabs), and GeneRead rRNA Depletion Kit with Globin mRNA Depletion Probes (Qiagen). Each kit was performed according to the manufacturer’s protocol.

### 2.10. Whole Transcriptome Amplification

For the “WTA/TNA” method, REPLI-g WTA Single Cell Kit (Qiagen) was used to perform first strand cDNA generation and whole transcriptome amplification (WTA) with 7 µL of concentrated TNA, according to the protocol for “Amplification of Purified RNA” [22]. Following WTA, PCR clean-up was performed using Agencourt AMPure XP beads (Beckman Coulter, Brea, CA, USA) [22]. For the “WTA DNase +/−” method, we performed the REPLI-g WTA Single Cell Kit “Amplification of Purified RNA” protocol twice on each sample: one-half of the sample volume was processed exactly according to protocol; the other half without DNAse treatment.

### 2.11. Quantification and Pooling

The resulting WTA products and the retained concentrated TNA were each quantitated with the Qubit dsDNA HS Assay Kit for the Qubit Fluorometer (Thermo Fisher Scientific, Waltham, MA, USA). Purified WTA products and TNA samples were each adjusted to 0.2 ng/µL and then pooled (2.5 µL each).

### 2.12. Library Preparation and Next-Generation Sequencing

Pooled WTA/TNA products (5 µL) were used to prepare the sequencing library with the Nextera XT DNA Library Preparation kit and Nextera XT Index Kit (Illumina, San Diego, CA, USA). Indexed libraries were purified with Agencourt AMPure XP beads (Beckman Coulter) [23]. Next-generation sequencing was performed using Illumina MiSeq, 300 cycle MiSeq Reagent Kit v2, and standard-sized flow cells (Illumina). Libraries were sequenced using either 2 × 75 or 2 × 151 paired-end protocols.

### 2.13. Data Analysis

During the initial sample preparation workflow development, sequence data (FASTQ files) from each sample were imported into CLC Bio Genomics Workbench (Qiagen) as paired-end reads accompanied by quality scores. Overlapping paired reads were merged, and the merged and unmerged reads were trimmed based on quality and length. Trimmed reads were mapped to a sequence list containing reference genomes for all target organisms and references for the human mitochondrial genome. Following read mapping analysis, data were subjected to additional analysis with the Kraken bioinformatics pipeline [24] (Baltimore, MD, USA).

FASTQ data files were also analyzed using the PanGIA bioinformatics pipeline [25] (Gaithersburg, MD, USA) at the command line using default settings for 2 × 75 base pair, paired-end Illumina reads. Data were exported to Microsoft Excel. The PanGIA bioinformatics pipeline can be accessed at https://github.com/mriglobal/PanGIA (accessed on 23 January 2023). The following data metrics were analyzed for this study: RNR, confidence score, background score, linear coverage, and depth of coverage. RNR (Reads Normalized by References) is the number of raw reads mapping to an individual reference genome divided by (normalized by) the number of other references it maps to. Standalone confidence score approximates how likely an organism is in a sample. This value is derived from a Bayesian probability distribution associated with the depth-of-coverage over the reference genome and the ‘uniqueness’ of the genomic loci that are covered. Background score is calculated by the ‘intersection over the union’ of coverage statistics for a particular species between a “background/negative control” and a target sample. Linear coverage is the percentage of the reference genome covered by mapped reads. Depth of coverage is the average number of reads that are mapped to a reference genome at a given nucleotide position. For comparison of data sets, we performed statistical analysis with a *t*-test to determine variance between two sample sets.

## 3. Results

### 3.1. Final Workflow Development Overview

**Figure 2:**
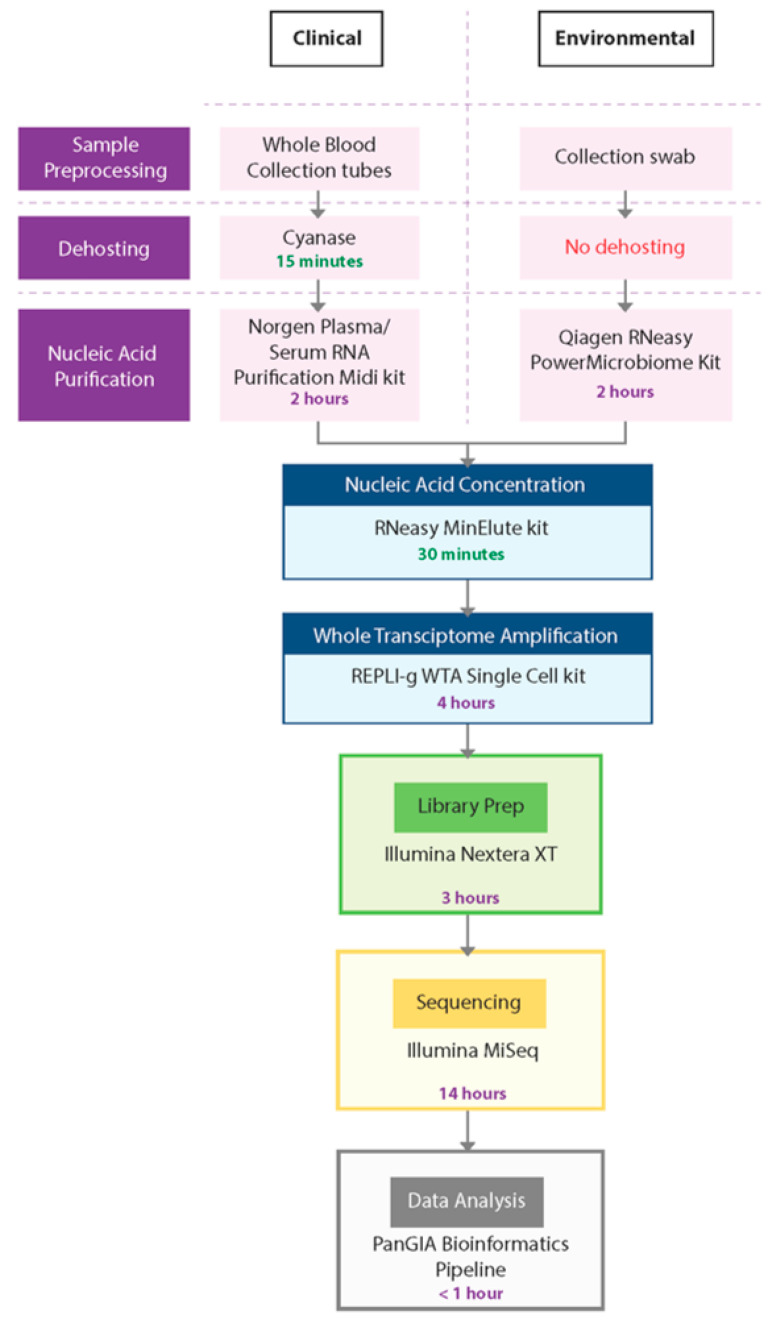
PanGIA Clinical and Environmental Workflows. This figure provides an illustration of the PanGIA Clinical and Environmental Workflows. The workflows share similar steps following Nucleic Acid Purification.

In this study, we developed a sample-to-sequence workflow for unbiased, metagenomic analysis of samples using NGS and an optimized bioinformatics pipeline to detect human pathogens. The evaluation, testing, and optimization of commercially available technologies resulted in the development of two separate workflows—the PanGIA Clinical Workflow and the PanGIA Environmental Workflow (Figure 2).

We optimized the PanGIA Clinical Workflow for the analysis of whole blood samples. Removing most red blood cells is a necessary dehosting step while retaining a small portion of RBCs that may contain intracellular pathogens. Following fractionation, the blood sample is further dehosted with the endonuclease Cyanase, which removes free-circulating nucleic acids from the sample before pathogen lysis. Next, the Plasma/Serum RNA Purification Kit (Norgen Biotek) purifies both RNA and DNA from dehosted, fractionated blood samples. We incorporated a bead-beating step with this kit to improve the lysis of difficult-to-lyse pathogens, such as gram-positive bacteria. Following nucleic acid purification, the RNeasy MinElute Cleanup kit further concentrates samples prior to whole transcriptome amplification (WTA).

To enable the detection of both DNA and RNA by sequencing, we developed the WTA/TNA approach. This approach utilizes the REPLI-g WTA Single Cell kit to generate whole transcriptome products from half of the concentrated sample while amplifying low titer targets from the raw extract (total nucleic acids, TNA). Using both the WTA product and concentrated TNA as inputs into library preparation, we could generate libraries from both DNA and RNA for sequencing with the Nextera XT DNA Library Prep kit. For the sequencing run, we chose a 2 × 75 paired-end (PE) protocol on the Illumina MiSeq platform.

The PanGIA Environmental Workflow is identical to the Clinical Workflow, except for the Nucleic Acid Purification kit and the absence of a dehosting step. We developed the PanGIA Environmental Workflow for surface collection swabs. The swabs are processed directly with the Qiagen RNeasy PowerMicrobiome kit for purification of both RNA and DNA. The purified nucleic acids are then processed through the remaining steps of the workflow for sequencing and data analysis. The following sections detail every step in the workflow development and the reasons we selected these technologies for incorporation into the final iteration of the workflow.

### 3.2. Dehosting of Clinical Samples Using Cyanase

It is well known that the sensitivity of metagenomic NGS relies on the ability to detect pathogen nucleic acids that are often masked by the high abundance of host nucleic acids [18]. Several reports indicate that removing host nucleic acids (dehosting) can improve the sensitivity of NGS-based methods for pathogen detection [26,27]. However, the extreme differences in the relative quantities of host versus pathogen genetic material in clinical matrices present a significant challenge. In this assessment, we categorized the dehosting methods as pre-lysis or post-purification host depletion methods. Pre-lysis host depletion methods include those that eliminate free-circulating host nucleic acids or host cells prior to pathogen lysis. Post-purification depletion methods include those that enrich pathogen-specific nucleic acids or deplete host-specific nucleic acids. We sought to determine: (1) which approach was most effective and (2) whether pre-lysis and post-purification methods could be used on the same sample for greater host reduction.

To deplete host nucleic acids before extraction, we fractionated whole blood samples. Before fractionation, we removed 25 µL of whole blood to reserve for extraction to detect intracellular pathogens while reducing the host background (in the form of red blood and white blood cells) significantly. Following fractionation, the plasma layer was separated from the buffy coat and RBS and combined with the 25 μL aliquot of whole blood.

We further evaluated pre-lysis depletion methods including differential lysis of blood cells with reagents such as EL Buffer (Qiagen) and Saponin. The most promising technology compatible with all our spiked targets was endonuclease digestion of free-circulating nucleic acids with enzymes such as Cyanase or Omnicleave. Further testing of the endonuclease reagents determined that Cyanase provided better pathogen detection over Omnicleave (Table 2). In addition, the Cyanase reagent includes an inactivation resin to stop residual nuclease activity following the release of pathogen nucleic acids.

**Figure 3:**
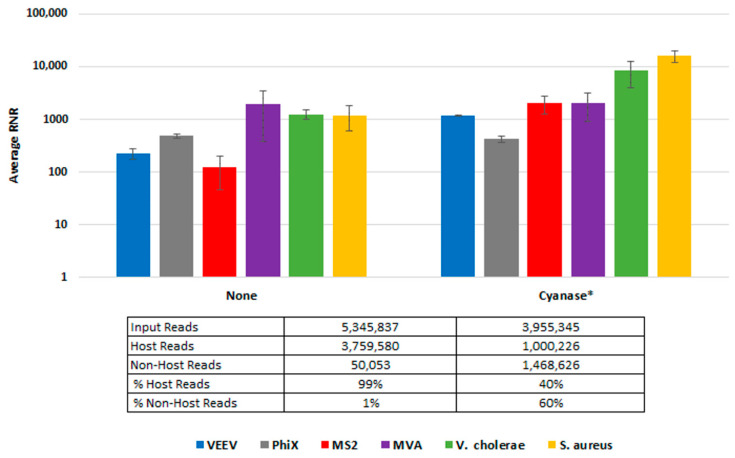
Cyanase Impact on Number of Reads. This figure compares average RNR values for fractionated blood samples treated with Cyanase and those without treatment. The final PanGIA Clinical Workflow was used to process these samples following extraction, with the sole difference limited to utilizing the Cyanase treatment or not. * Indicates only three samples included in the data set, instead of four (one sample was excluded due to a low number of input reads).

To demonstrate Cyanase dehosting in whole blood, we spiked samples with *S. aureus*, MVA virus, VEE virus, and *V. cholerae*. Figure 3 shows the RNR for each species in the presence or absence of Cyanase. In the presence of Cyanase, there is an approximately 10-fold increase in the RNR value for *S. aureus*, VEE virus, and *V. cholerae*, while there is no difference in RNR for the MVA virus. Figure 3 shows the number (and percentage) of host and non-host reads detected in the presence or absence of Cyanase. In this study, there was an average reduction of 2.76 million (58.7%) host reads and an average increase of 1.42 M non-host reads, up from an average of 1.3% non-host reads without Cyanase to 60% non-host reads with Cyanase.

**Figure 4:**
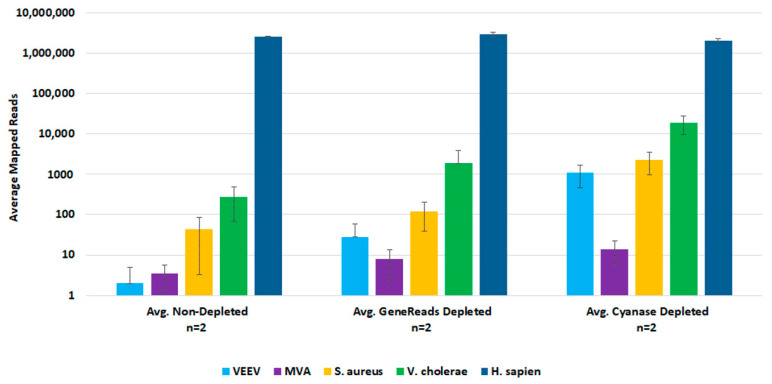
Comparison of Pre-lysis and Post-purification Host Depletion Methods. We evaluated both pre-lysis and post-purification host depletion methods with replicate samples. Sequencing data was analyzed in CLC Bio Genomics Workbench. The average number of mapped reads were used to evaluate target organism detection.

For post-lysis host depletion, there were several kits available to enrich microbial DNA and/or ribosomal RNA depletion. We evaluated the NEBNext Microbiome DNA Enrichment kit, the NEBNext rRNA Depletion kit, and the GeneReads rRNA depletion kit (with globin mRNA depletion probes) with wet-lab experiments. Initial testing with the NEBNext kits confirmed that post-lysis host depletion results in improved detection of target pathogens. However, the Microbiome DNA enrichment kit resulted in the loss of viral target detection, and the rRNA depletion kit prevented the detection of the DNA viruses. We evaluated the GeneReads rRNA depletion kit with samples that had also been dehosted with Cyanase. However, Cyanase treatment resulted in samples with total RNA quantities unsuitable for performing GeneReads depletion. Comparison testing of pre-lysis Cyanase treatments versus post-purification GeneReads dehosting showed that the pre-lysis host depletion with Cyanase resulted in better detection of pathogen nucleic acids (Figure 4).

### 3.3. Extraction of Total Nucleic Acids

We evaluated several nucleic acid extraction and purification kits independent of our dehosting testing, including kits designed for cell-free circulating nucleic acids and preserved blood RNA kits. Our initial testing identified the Qiagen QIAamp DNA Blood kit and the Norgen Plasma/Serum RNA Isolation kit as the two best-performing kits (Table S1). Both kits provided the best qPCR detection of our spiked pathogens and thus were selected for further testing.

**Figure 5:**
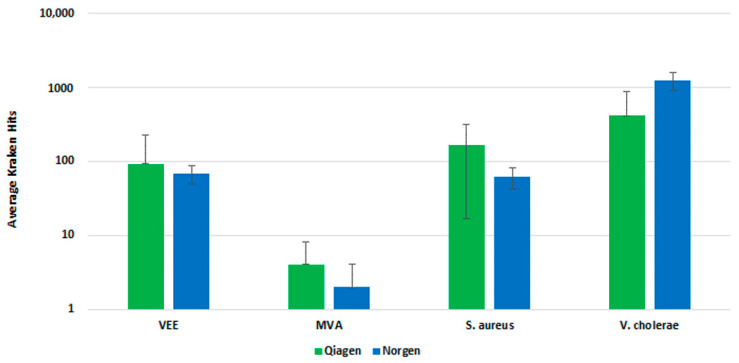
Clinical Extraction Kit Comparison. This figure illustrates the average number of Kraken hits for n = 3 replicate extraction reactions with the Qiagen and Norgen extraction kits.

We further evaluated the Qiagen and Norgen kits with fractionated whole blood sample input and a pre-lysis host depletion step (Omnicleave). In a side-by-side comparison, both kits performed similarly, demonstrating a similar number of Kraken hits upon analysis (Figure 5). The Qiagen kit generated a higher average number of Kraken hits than the Norgen kit, but there was less standard deviation with the replicates for the Norgen kit. Ultimately, we selected the Norgen kit for inclusion in the final workflow, due to the protocol’s ease of use and compatibility with the fractionation and pre-lysis host depletion steps.

**Figure 6:**
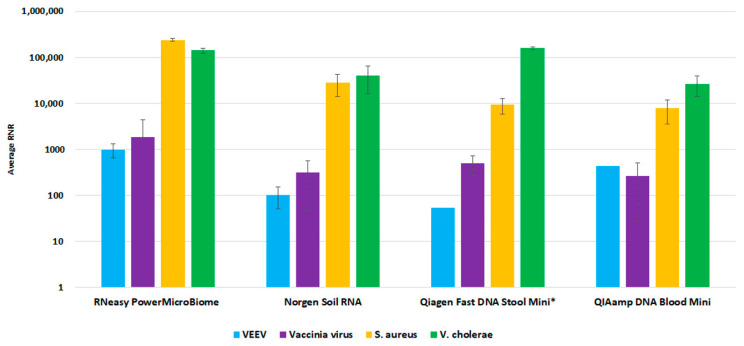
Environmental Extraction Kit Comparison. This figure illustrates average RNR values for four pathogen surrogates (VEE virus, Vaccinia virus, *S. aureus*, *V. cholerae*) for four different extraction kits.

For the environmental workflow, fewer commercial total nucleic acid available kits were available for consideration. We performed a side-by-side comparison of four extraction kits, Qiagen RNeasy PowerMicrobiome kit, Norgen Soil Total RNA Purification kit, Qiagen QIAamp Fast DNA Stool Mini Kit, and Qiagen QIAamp DNA Blood Mini kit. The RNeasy PowerMicrobiome kit outperformed the other evaluated kits, with a higher average RNR value for viral target detection relative to the other kits (Figure 6). Thus, we incorporated this kit into our workflow as the preferred extraction method for environmental swab samples.

### 3.4. Concentrating Total Nucleic Acids

Nucleic acid purification resulted in a sample volume too large to allow the entire sample to be used for cDNA conversion and library preparation. For samples with low titer of pathogens, this could result in reduced detection sensitivity. We evaluated the Qiagen RNeasy MinElute Cleanup Kit, and the Norgen RNA Clean-Up and Concentration Micro-Elute Kit to concentrate purified TNA samples. To ensure equal amounts of TNA were purified using both kits, extracts were pooled prior to purification and concentration with the Norgen Micro-Elute kit or the Qiagen MinElute kit. WTA and library preparation was then performed on unconcentrated TNA or TNA concentrated with either MinElute or Norgen kits. The use of the concentrated extract, regardless of the kit, resulted in increased detection (RNR) of spiked targets (Table 3). However, we selected the Qiagen RNeasy MinElute Cleanup kit due to consistently higher RNR, linear coverage and depth of coverage for most of the spiked targets, with *S. aureus* being the only exception.

### 3.5. Whole Transcriptome Amplification

**Figure 7:**
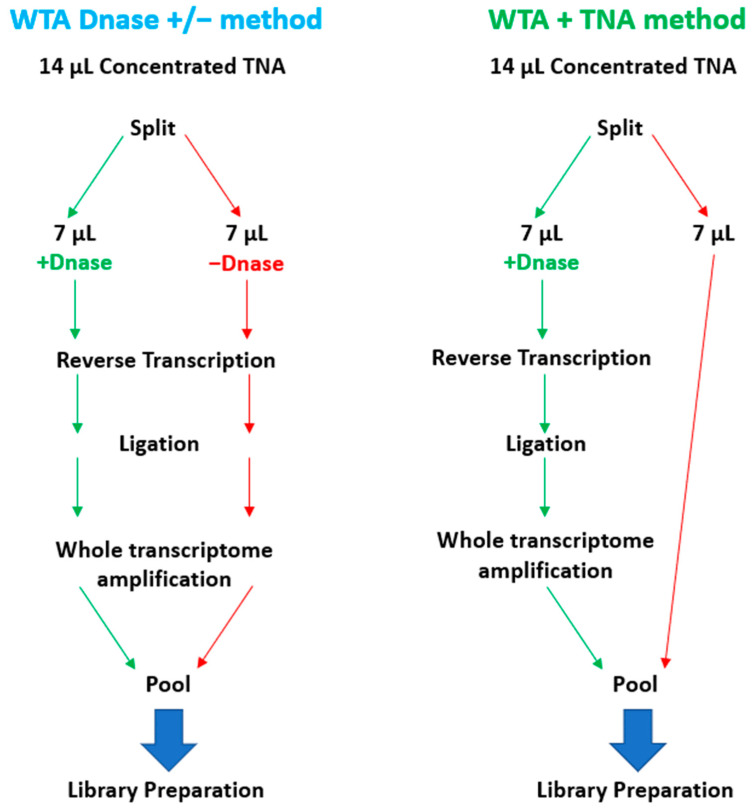
Schematic Diagram of WTA Methods Evaluated in this Study. This figure illustrates the two WTA methods that were under consideration for integration into the PanGIA workflow. The WTA & TNA method was ultimately selected for incorporation into the final iteration of the PanGIA workflow.

To enable the sequencing of RNA targets on the Illumina MiSeq, it is necessary to convert the RNA to cDNA before library preparation. To accomplish this, RNA can be converted to cDNA by reverse transcription or whole transcriptome amplification (WTA). WTA enables sensitive detection of RNA targets since the method includes both a reverse transcription and an amplification step. We selected the Qiagen REPLI-g WTA Single Cell kit based on ease of use and a relatively short turnaround time of ~4 h. However, the standard WTA protocol includes using a DNase, which removes genomic DNA (gDNA) before reverse transcription. For unbiased pathogen detection, capturing both DNA and RNA targets in the same sample is essential. To enable the detection of DNA targets, we split the 14 µL total nucleic acid (TNA) sample and processed one-half through the manufactured recommended protocol and reserved the remaining half for the detection of DNA targets. We refer to this as the WTA & TNA method, as shown in Figure 7. We had previously processed two 7 µL volumes of the TNA sample through the WTA protocol both in the presence and absence of DNase (WTA DNase ± method).

**Figure 8:**
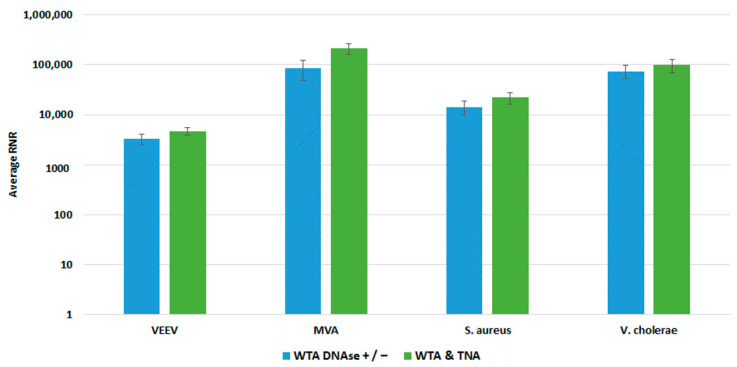
Comparison of WTA Methods, Clinical Workflow. This figure compares RNR values for spiked targets in samples prepared from two different WTA methods. Sequencing libraries prepared from combining equal volumes of two normalized WTA reactions, one with and one without a DNase step, are referred to as WTA DNase +/−. Sequencing libraries prepared by combining equal volumes of normalized WTA and TNA are referred to as WTA & TNA.

**Figure 9:**
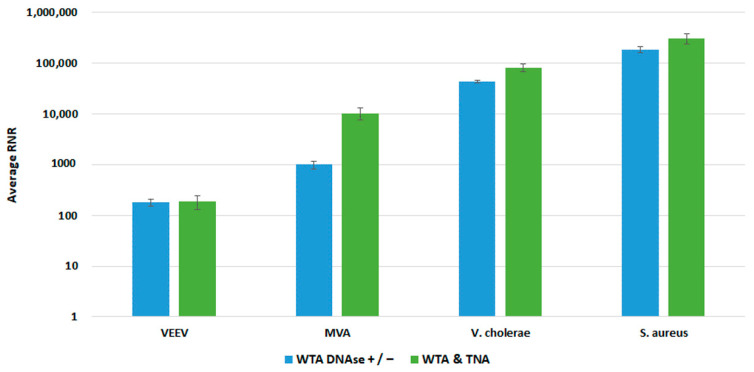
Comparison of WTA Methods, Environmental Workflow. This figure compares RNR values for spiked targets in samples prepared from two different WTA methods. Sequencing libraries prepared from combining equal volumes of two normalized WTA reactions, one with and one without a DNase step, are referred to as WTA DNase +/−. Sequencing libraries prepared from combining equal volumes of normalized WTA and TNA are referred to as WTA & TNA.

We compared both WTA approaches with our clinical and environmental sample preparation workflows, as shown in Figure 8 and Figure 9, respectively. In most cases, there was no statistically significant difference in detection of our targets with whole blood. For Vaccinia, we noted a statistically significant 2-fold increase in RNR value for the clinical workflow. There was a statistically significant increase in RNR for the environmental workflow with the WTA & TNA Method versus the WTA DNase ± method for all targets, except for VEE virus. Given these findings, we incorporated the WTA & TNA method into our workflow to reduce unnecessary sample handling and reagent costs.

### 3.6. Sequencing Protocol Impact on Pathogen Detection Sensitivity and Specificity

**Figure 10:**
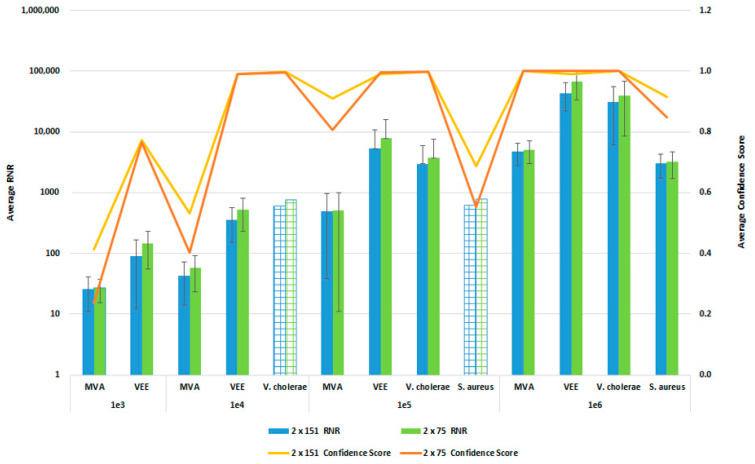
Sequencing Run Comparison: Whole Blood Samples. This figure summarizes the average RNR and Confidence Scores based on 4 of 4 contrived, whole blood replicate samples. Bars represent sample RNR values and lines represent sample confidence scores. Bars with grid lines indicate 1 of 4 replicates detected. For this analysis, we utilized notional LoD criteria of a minimum RNR value of 100 and a Confidence Score of 0.5.

**Figure 11:**
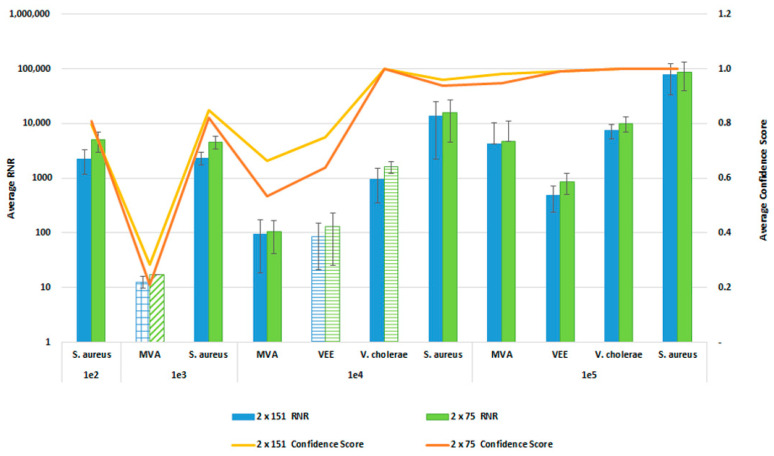
Sequencing Run Comparison: Environmental Samples. This figure summarizes the average RNR and Confidence Scores based on 4 of 4 contrived, RBM swab replicate samples. Bars represent sample RNR values and lines represent sample confidence scores. Bars with horizontal lines indicate 3 of 4 replicates detected. Bars with diagonal indicate 2 of 4 replicates detected. Bars with grid lines indicate 1 of 4 replicates detected. For this analysis, we utilized notional LoD criteria of a minimum RNR value of 100 and a Confidence Score of 0.5.

In order to reduce the turnaround time from sample-to-answer, we investigated the use of a 2 × 75 paired end sequencing run versus the 2 × 151 paired-end protocol that we used during the initial development of the workflow. While the 2 × 75 paired-end protocol provides a reduction in 12 h of sequencing time, we were concerned about the potential for reduced detection sensitivity and specificity with the shorter sequencing run due to the potential impact on resulting confidence scores. For this comparison, whole blood was co-spiked with internal positive controls (MS2 and PhiX bacteriophages), and four pathogens, *S. aureus*, MVA virus, VEE virus, and *V. cholerae*. All four pathogens were co spiked into 1 mL of whole blood at serial dilutions of 1 × 10^6^, 1 × 10^5^, 1 × 10^4^, or 1 × 10^3^ CFU/PFU per mL. Four samples for each spike level were processed using the PanGIA Clinical Workflow and ran using a 2 × 75 or 2 × 151 sequencing run. Using statistical analysis, we determined that there was no significant difference in the RNR values and confidence scores between the sequencing run parameters for these samples (Figure 10). This is true for the Environmental Workflow as well, where we co-spiked internal positive controls with four pathogen surrogates at 1 × 10^5^, 1 × 10^4^, 1 × 10^3^, and 1 × 10^2^ CFU/ PFU (Figure 11). Therefore, we chose 2 × 75 sequencing runs as the standard protocol, which saves approximately 12 h of processing time and enables a sample-to-answer in under 24 h. Additional data for all other spike levels can be found in Tables S2and S3. Based on the acceptance criteria of a minimal average RNR score of 100 and an average Confidence Score of 0.5 for all four technical replicates, we were able to determine notional Limits of Detection (LoD) for our target organisms. We calculated LoDs with the clinical workflow of 1 × 10^5^ CFU, 1 × 10^6^ CFU, 1 × 10^5^ PFU and 1 × 10^4^ PFU for *V. cholerae*, *S. aureus*, MVA virus, and VEE virus, respectively (Figure 10). For the environmental workflow, we detected all four targets at 1 × 10^5^ CFU or PFU (Figure 11).

### 3.7. Inclusivity Testing of Clinical and Environmental Workflows

**Figure 12:**
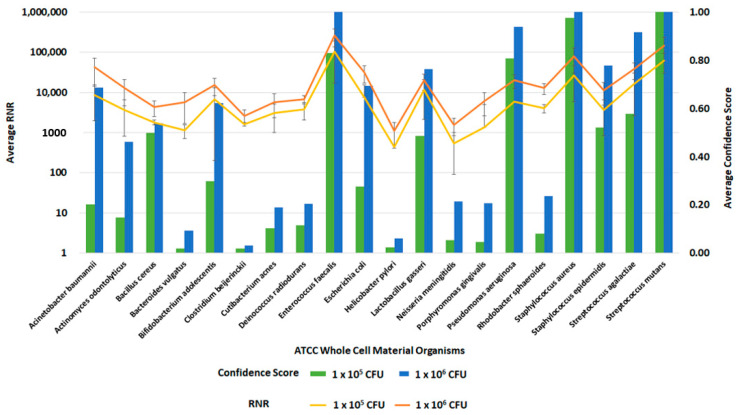
Inclusivity of ATCC Whole Cell Material Microbiome Standard—Whole Blood. This figure summarizes the average RNR and Confidence Scores based on 4 of 4 contrived, whole blood replicate samples. Bars represent sample confidence scores, and lines represent sample RNR values. At 1 × 10^5^ CFU/ mL, the following were detected with less than 4 replicates: *C. acnes* (3), *P. gingivalis* (3), *B. vulgatus* (2), *C. beijerinckii* (2), *N. meningitides* (2), *R. sphaeroides* (2), *H. pylori* (1), *P. aeruginosa* (1).

**Figure 13:**
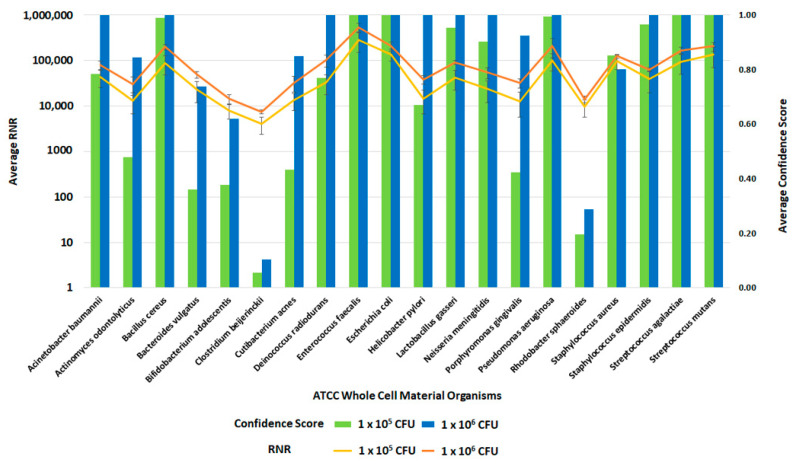
Inclusivity of ATCC Whole Cell Material Microbiome Standard—RBM Swabs. This figure summarizes the average RNR and Confidence Scores based on 4 of 4 contrived, RBM swab replicate samples. Bars represent sample confidence scores, and lines represent sample RNR values.

Following the optimization of the clinical and environmental workflows, we sought to verify the system’s general ability to detect pathogens. For this evaluation, we spiked whole blood and RBM swabs with the ATCC 20 Strain Even Mix Whole Cell Material (ATCC MSA-2002), a microbiome standard that consists of 20 gram-positive and gram-negative bacteria in equal concentrations. Clinical and environmental samples were spiked with MSA-2002 at concentrations of 1 × 10^6^, 1 × 10^5^ or 1 × 10^4^ CFU of each organism and processed using the respective workflows. The 1 × 10^6^ and 1 × 10^5^ CFU organism results of these studies are presented in Figure 12 and Figure 13. Testing the microbiome standard enabled the evaluation of a diverse mixture of 20 gram-positive and gram-negative bacteria. Figure 12 and Figure 13 demonstrate the detection of reads for all twenty organisms in whole blood and RBM, respectively. We noted a difference in performance of the microbiome standard in our two sample matrices as it related to RNR and confidence score values. Overall, the confidence scores for the twenty organisms were much lower in whole blood versus the RBM swabs. This difference in performance may be due to the high-host background nature of clinical samples. These results show that the presence of host genomic material affects the metagenomic detection sensitivity of pathogens.

## 4. Discussion

We developed and optimized a standardized workflow for unbiased pathogen detection from clinical or environmental samples in this study. Here, we present data from developmental and analytical validation studies for the PanGIA clinical and environmental workflows. Several NGS methods are available in the literature. However, most published methods are limited by targeting a specific pathogen class [28], sample matrix, or require several days for processing and analysis of all pathogen targets [18,29,30,31]. To the best of our knowledge, this is the first report of a standardized NGS-based end-to-end solution for the unbiased detection of pathogens (bacterial and viral) from clinical or environmental samples in under 24 h. We optimized this workflow for detection sensitivity by incorporating endonuclease dehosting, bead-beating lysis, and total nucleic acid concentration into the protocol. The WTA & TNA approach provides a novel solution for the simultaneous NGS analysis of DNA and RNA from metagenomic samples. We confirmed that a 2 × 75 PE protocol provides the similar sensitivity as a 2 × 150 PE protocol, which was crucial for ensuring a 24-h turnaround time.

Selection of a host depletion method was a challenging aspect of the clinical workflow development. A recent review of metagenomic NGS methods for infectious disease diagnosis found several clinical cases that resulted in successful diagnosis of pathogens from less than 1000 mapped sequencing reads [32]. Thus, a significant reduction in the number of host reads, which are typically in the millions, should increase the number of detected pathogen reads. We evaluated several technologies that included pre-lysis and post-purification methods (Figure 1). Ultimately, we determined that pre-lysis endonuclease digestion of free-circulating nucleic acids with Cyanase was the most suitable for our workflow (Table 2).

To determine any potential loss of detection sensitivity and specificity due to reducing our sequencing protocol from a 2 × 151 paired-end run to 2 × 75 paired-end protocol, we performed studies using a serial dilution of our four pathogen surrogates (*V. cholerae*, *S. aureus*, MVA virus and VEE virus), representative of a gram-negative bacteria, gram-positive bacteria, DNA, and RNA virus, respectively. The data from these analyses indicate that there was no loss of detection sensitivity, as the RNR values and the Confidence Scores remained similar between the two sequencing protocols. At concentrations below the observed LoD, we detect reads for the spiked targets; however, the low abundance (low depth of coverage) and diversity (low linear coverage) results in a low confidence score. The LoDs observed here are 1-2-log higher than typical qPCR methods, such as the FilmArray Meningitis/Encephalitis (ME) Panel, with observed LoDs of 50 500 CFU/PFU per mL [33]. However, the PanGIA LoDs are more comparable to those observed with some recent targeted enrichment NGS methods [34]. While the detection sensitivity is not equivalent to blood culture methods, this workflow would detect intact, dead cells in blood that would not be viable in culture, while offering a significant improvement in turnaround time. In addition, pathogen targets with low confidence scores could still inform follow-on targeted detection opportunities. Further optimization of the workflow will be required to improve detection sensitivity prior to integration into a clinical laboratory.

The criteria for LoD determination that we provide in this paper serves the purpose of providing an approximate analytical sensitivity and to establish a threshold for our analysis. Further analytical validation of individual organisms and sample matrices will need to be performed to determine true LoDs. However, the discovery power of the PanGIA bioinformatics pipeline is one of the true benefits of this tool. It provides the user with the ability to look further into the data beyond the read count of target pathogens in a sample and to utilize the various confidence scores to better understand the data. For example, with our environmental workflow, we observed similar RNR values and Confidence Scores for *S. aureus* at both 1 × 10^3^ and 1 × 10^3^ CFUs. To resolve this apparent discrepancy, we compared the average Background score for these dilution points. The Background Score indicates how closely a sample composition compares to a negative sample matrix (processing control). The Background Scores for these samples suggest that these reads originate from the sample matrix background, given the low values (0.01) for this metric. The Background Score increases for the 1 × 10^5^ CFU spike, indicating actual detection of *S. aureus* beyond what is inherent to the sample background.

Future work will focus on improving the efficiency and sensitivity of the PanGIA workflows, through continued gap analysis to identify and evaluate new technologies, as they become available. While the data presented in this work illustrates the importance of Cyanase as a dehosting method, a substantial amount of host nucleic acids remain in clinical samples. To address host background reduction, we are interested in technologies that enrich libraries prior to sequencing, such as probes for removing human genomic material [35]. In addition, we will continue efforts to expand the workflows to include other relevant clinical and environmental matrices, such as serum, nasopharyngeal swabs, mosquitoes, etc., as well as additional classes of pathogens (eukaryotic pathogens and parasites). Finally, future work will include optimization for sample analysis in austere conditions, such as mobile laboratories or other field-forward settings. This capability would allow the workflows to be used to their full potential—providing unbiased pathogen detection, in less than 24 h, in settings where traditional methods would not be feasible or sufficient.

## 5. Conclusions

The development of a universal method for pathogen detection from sample to answer requires overcoming several challenges including, but not limited to, addressing bacterial versus viral extraction methods, purification of both DNA and RNA, reduction in host nucleic acids and concentration of low-level pathogens. The work presented here is a standardized solution toward addressing these needs for infectious disease pathogen detection and discovery. The PanGIA workflows were optimized to increase end users’ ease-of-use, while minimizing hands-on processing time, to allow for a 24-h turnaround from sample-to-answer. The workflow incorporates novel processes for dehosting and simultaneous sequencing of RNA and DNA targets. We have developed standardized operating procedures that will be open access for end users to integrate into their pathogen detection protocols. In summary, this work decreases the barrier of entry for laboratories looking to adopt metagenomic biosurveillance capabilities into their routine work.

## Figures and Tables

**Table 1:** Sample-to-Sequence Operational Requirements. This table summarizes the sponsor-specified operational requirements that were taken into consideration during the development of the PanGIA workflow.

Requirement
Compatible with mobile laboratories; standardized operating procedures (SOPs); utilization of COTS reagents
Sample-to-answer, including data analysis, within 24 h
Universal sample preparation workflow to enable detection of all pathogen types, including bacteria and viruses
Development of a straightforward, “push-button” bioinformatics workflow using commodity hardware

**Table 2:** Comparison of Pre-Lysis Host Depletion Methods. Host depletion methods were evaluated on nucleic acids extracted with the Norgen Plasma/ Serum RNA kit. Detection of target organisms was determined by comparing performance metrics; RNR, Confidence Score, Linear Coverage and Depth of Coverage. Data for Vaccinia virus was not available for this study.

Analysis Metric	Target Organism	None n = 4	Cyanase n = 4	Omnicleave n = 5
RNR	*S. aureus*	185.9	1337.7	-
	VEE virus	33.0	306.0	39.5
	*V. cholerae*	1711.0	7219.8	1229.8
Confidence Score	*S. aureus*	0.47	0.84	-
	VEE virus	0.44	0.99	0.51
	*V. cholerae*	1.00	1.00	1.00
Linear Coverage	*S. aureus*	1%	1%	-
	VEE virus	26%	69%	29%
	*V. cholerae*	4%	5%	3%
Depth of Coverage	*S. aureus*	0.026	0.151	-
	VEE virus	0.043	3.965	0.513
	*V. cholerae*	1.062	2.640	0.697

**Table 3:** Comparison of Concentration Kits (Norgen versus Qiagen). This table represents average results from replicates (n = 4) of each nucleic acid concentration method. We assessed the detection of target organisms by comparing performance metrics; RNR, Confidence Score, Linear Coverage and Depth of Coverage.

Species	Concentration Method	Average RNR	Average Confidence Score	Average Linear Coverage	Average Depth of Coverage
PhiX	None	1391	1.00	0.90	18.91
	MinElute	2944	0.99	1.00	40.29
	Norgen	1908	0.99	0.97	26.08
MS2	None	327	0.99	0.65	6.76
	MinElute	8915	0.99	0.92	184.71
	Norgen	6309	0.99	0.91	130.53
*S. aureus*	None	4102	0.82	0.01	0.21
	MinElute	19,163	0.97	0.01	1.07
	Norgen	21,715	0.97	0.02	1.34
VEEV	None	3337	1.00	0.92	21.61
	MinElute	243,033	1.00	0.99	1573.63
	Norgen	225,427	1.00	0.99	1459.31
*V. cholerae*	None	10,024	1.00	0.08	2.66
	MinElute	40,891	1.00	0.20	10.56
	Norgen	33,018	1.00	0.14	8.57

## Data Availability

The data presented in this study are available on request from the corresponding author.

## Supplementary Materials

The following supporting information can be downloaded at: https://www.mdpi.com/article/10.3390/tropicalmed8020121/s1, Table S1: PCR Analysis of Candidate Extraction Methods; Table S2: Clinical Workflow LOD; Table S3: Environmental Workflow LOD.
