# Supplementary material for: Development and Optimization of an Unbiased, Metagenomics-Based Pathogen Detection Workflow for Infectious Disease and Biosurveillance Applications
Source: Trop Med Infect Dis. 2023 Feb 15;8(2):121. doi: 10.3390/tropicalmed8020121 (PMC9966482; doi:10.3390/tropicalmed8020121)
Supplement: Supplementary file 1 [file tropicalmed-08-00121-s001.zip › tropicalmed-2178433-supplementary.pdf]

Table S1. PCR Analysis of Candidate Extraction Methods.

We evaluated the performance of candidate extraction methods by real-time PCR for detection of spiked targets in whole blood. Extraction kits were screened for further analysis. We also evaluated the analysis of spiked whole blood that had been fractionated, as described in the Methods section. Ct values presented are the average of 2 real-time PCR replicates per sample extract replicate. Values in italics indicates samples when a single real-time PCR replicate was detected. N/D = no detection.

		Whole Blood Avg. Ct values (n=2)						Fractionated Blood Avg. Ct values (n=2)	
	Rep#	QIAamp DNA Blood Maxi Kit	PAXgene Blood RNA System	Norgen Preserved Blood RNA Purification Kit	RNAgard Blood System	NucleoSpin Blood RNA Midi	E.Z.N.A. Blood RNA Midi kit	QIAamp DNA Blood Maxi Kit	Norgen Plasma/Serum RNA Maxi Kit
S. aureus	1	33.0	*37.0*	36.8	38.5	*38.9*	N/D	33.1	32.7
	2	33.2	*38.4*	37.8	40.0	38.2	*39.6*	33.3	33.1
Vaccinia virus	1	40.4	41.5	43.6	49.6	N/D	42.8	40.5	45.1
	2	41.2	41.7	45.2	N/D	N/D	42.3	41.2	39.4
V. cholerae	1	33.0	*39.0*	35.0	40.5	N/D	N/D	36.8	35.3
	2	33.3	38.7	35.9	N/D	N/D	N/D	36.6	35.5
VEE virus	1	31.3	*33.4*	38.6	Data not collected		38.2	34.4	30.9
	2	31.3	33.5	40.0			37.1	33.6	31.1

Table S2. Clinical Workflow LoD.

We determined the LoD of our target organisms with the PanGIA Clinical Workflow based on the following data analysis performance metrics: RNR, Linear Coverage, Depth of Coverage, Confidence Score and Background Score. The table below summarizes the average and standard deviation values of these performance metrics.

Species	Spike Level	RNR		Linear Coverage		Depth of Coverage		Confidence Score		Background Score	
		2x75	2x151	2x75	2x151	2x75	2x151	2x75	2x151	2x75	2x151
Staphylococcus aureus	1e5	777 ± 0	627 ± 0	0.01 ± 0.00	0.01 ± 0.00	0.04 ± 0.00	0.08 ± 0.00	0.55 ± 0.00	0.69 ± 0.00	1.00 ± 0.00	1.00 ± 0.00
	1e6	3,196 ± 1515	3,054 ± 1289	0.01 ± 0.00	0.01 ± 0.00	0.21 ± 0.08	0.47 ± 0.17	0.85 ± 0.05	0.92 ± 0.05	1.00 ± 0.00	1.00 ± 0.00
Vaccinia virus	1e3	27 ± 11	26 ± 15	0.02 ± 0.01	0.02 ± 0.01	0.02 ± 0.01	0.03 ± 0.02	0.24 ± 0.08	0.41 ± 0.18	1.00 ± 0.00	1.00 ± 0.00
	1e4	57 ± 34	43 ± 29	0.03 ± 0.02	0.03 ± 0.02	0.04 ± 0.03	0.05 ± 0.04	0.40 ± 0.12	0.53 ± 0.13	1.00 ± 0.00	1.00 ± 0.00
	1e5	510 ± 499	497 ± 458	0.20 ± 0.14	0.21 ± 0.14	0.38 ± 0.38	0.54 ± 0.49	0.81 ± 0.13	0.91 ± 0.08	1.00 ± 0.00	1.00 ± 0.00
	1e6	5,105 ± 2064	4,692 ± 1855	0.65 ± 0.07	0.64 ± 0.08	3.90 ± 1.57	5.18 ± 2.06	1.00 ± 0.00	1.00 ± 0.00	1.00 ± 0.00	1.00 ± 0.00
Venezuelan equine encephalitis virus	1e3	144 ± 89	91 ± 78	0.42 ± 0.18	0.45 ± 0.19	0.93 ± 0.58	1.18 ± 1.01	0.76 ± 0.33	0.77 ± 0.34	1.00 ± 0.00	1.00 ± 0.00
	1e4	523 ± 292	361 ± 208	0.72 ± 0.13	0.78 ± 0.09	3.40 ± 1.89	4.67 ± 2.70	0.99 ± 0.00	0.99 ± 0.00	1.00 ± 0.00	1.00 ± 0.00
	1e5	7,805 ± 8259	5,286 ± 5500	0.96 ± 0.03	0.95 ± 0.05	50.55 ± 53.49	68.69 ± 71.49	1.00 ± 0.00	0.99 ± 0.00	1.00 ± 0.00	1.00 ± 0.00
	1e6	68,109 ± 34282	43,096 ± 21223	0.99 ± 0.00	0.99 ± 0.06	440.74 ± 221.80	558.97 ± 275.14	1.00 ± 0.00	0.99 ± 0.00	1.00 ± 0.00	1.00 ± 0.00
Vibrio cholerae	1e4	766 ± 0	598 ± 0	0.01 ± 0.00	0.01 ± 0.00	0.26 ± 0.00	0.42 ± 0.00	1.00 ± 0.00	1.00 ± 0.00	1.00 ± 0.00	1.00 ± 0.00
	1e5	3,722 ± 3811	2,982 ± 3008	0.03 ± 0.04	0.06 ± 0.06	1.21 ± 1.27	2.10 ± 2.22	1.00 ± 0.00	1.00 ± 0.00	1.00 ± 0.00	1.00 ± 0.00
	1e6	39,053 ± 30441	31,147 ± 25026	0.31 ± 0.22	0.39 ± 0.26	13.92 ± 13.31	23.05 ± 22.26	1.00 ± 0.00	1.00 ± 0.00	1.00 ± 0.00	1.00 ± 0.00

Table S3. Environmental Workflow LoD.

We determined the LoD of our target organisms with the PanGIA Environmental Workflow based on the following data analysis performance metrics: RNR, Linear Coverage, Depth of Coverage, Confidence Score and Background Score. The table below summarizes the average and standard deviation values of these performance metrics.

Species	Spike Level	RNR		Linear Coverage		Depth of Coverage		Confidence Score		Background Score	
		2x75	2x151	2x75	2x151	2x75	2x151	2x75	2x151	2x75	2x151
**Staphylococcus aureus**	1e2	4,988 ± 1,981	2,260 ± 1,076	0.01 ± 0.00	0.01 ± 0.00	0.40 ± 0.17	0.42 ± 0.21	0.81 ± 0.06	0.80 ± 0.07	0.00 ± 0.00	0.01 ± 0.00
	1e3	4,628 ± 1,149	2,349 ± 612	0.01 ± 0.00	0.01 ± 0.00	0.36 ± 0.08	0.41 ± 0.09	0.82 ± 0.03	0.85 ± 0.04	0.01 ± 0.00	0.03 ± 0.00
	1e4	15,634 ± 11,072	13,468 ± 11,238	0.01 ± 0.00	0.01 ± 0.00	0.92 ± 0.61	1.53 ± 1.21	0.94 ± 0.09	0.96 ± 0.08	0.01 ± 0.00	0.55 ± 0.52
	1e5	87,743 ± 47,423	78,149 ± 45,172	0.01 ± 0.01	0.02 ± 0.01	4.44 ± 2.12	7.72 ± 3.72	1.00 ± 0.00	1.00 ± 0.00	0.75 ± 0.50	1.00 ± 0.00
**Vaccinia virus**	1e3	17 ± 0	13 ± 3	0.01 ± 0.00	0.01 ± 0.00	0.01 ± 0.00	0.01 ± 0.00	0.21 ± 0.00	0.28 ± 0.03	1.00 ± 0.00	1.00 ± 0.00
	1e4	105 ± 64	95 ± 77	0.06 ± 0.03	0.07 ± 0.05	0.08 ± 0.05	0.10 ± 0.08	0.53 ± 0.17	0.66 ± 0.23	1.00 ± 0.00	1.00 ± 0.00
	1e5	4,640 ± 6,564	4,257 ± 5,932	0.50 ± 0.30	0.52 ± 0.28	3.45 ± 4.86	4.61 ± 6.40	0.95 ± 0.06	0.98 ± 0.03	1.00 ± 0.00	1.00 ± 0.00
**Venezuelan equine encephalitis virus**	1e4	129 ± 103	85 ± 64	0.36 ± 0.14	0.39 ± 0.14	0.83 ± 0.66	1.10 ± 0.82	0.64 ± 0.32	0.75 ± 0.21	1.00 ± 0.00	1.00 ± 0.00
	1e5	858 ± 355	479.5 ± 244	0.82 ± 0.07	0.79 ± 0.10	5.55 ± 2.29	6.21 ± 3.16	0.99 ± 0.00	0.99 ± 0.00	1.00 ± 0.00	1.00 ± 0.00
**Vibrio cholerae**	1e4	1,607 ± 372	940 ± 584	0.01 ± 0.00	0.01 ± 0.01	0.23 ± 0.16	0.30 ± 0.21	1.00 ± 0.00	1.00 ± 0.00	1.00 ± 0.00	1.00 ± 0.00
	1e5	10,093 ± 3051	7,377 ± 2174	0.05 ± 0.05	0.08 ± 0.08	1.75 ± 1.59	2.96 ± 2.78	1.00 ± 0.00	1.00 ± 0.00	1.00 ± 0.00	1.00 ± 0.00
